# A preliminary evaluation of a high temporal resolution data-driven motion correction algorithm for rubidium-82 on a SiPM PET-CT system

**DOI:** 10.1007/s12350-020-02177-2

**Published:** 2020-05-21

**Authors:** Ian S. Armstrong, Charles Hayden, Matthew J. Memmott, Parthiban Arumugam

**Affiliations:** 1 Nuclear Medicine, Manchester University NHS Foundation Trust, Oxford Road, Manchester, UK; 2 Molecular Imaging, Siemens Medical Solutions USA, Inc., Knoxville, TN USA

**Keywords:** PET, MPI, Image reconstruction, Image analysis, Image interpretation

## Abstract

**Background:**

In myocardial perfusion PET, images are acquired during vasodilator stress, increasing the likelihood of intra-frame motion blurring of the heart in reconstructed static images to assess relative perfusion. This work evaluated a prototype data-driven motion correction (DDMC) algorithm designed specifically for cardiac PET.

**Methods:**

A cardiac torso phantom, with a solid defect, was scanned stationary and being manually pulled to-and-fro in the axial direction with a random motion. Non-motion-corrected (NMC) and DDMC images were reconstructed. Total perfusion deficit was measured in the defect and profiles through the cardiac insert were defined. In addition, 46 static perfusion images from 36 rubidium-82 MPI patients were selected based upon a perception of motion blurring in the images. NMC and DDMC images were reconstructed, blinded, and scored on image quality and perceived motion.

**Results:**

Phantom data demonstrated near-perfect recovery of myocardial wall visualization and defect quantification with DDMC compared with the stationary phantom. Quality of clinical images was NMC: 10 non-diagnostic, 31 adequate, and 5 good; DDMC images: 0 non-diagnostic, 6 adequate, and 40 good.

**Conclusion:**

The DDMC algorithm shows great promise in rubidium MPI PET with substantial improvements in image quality and the potential to salvage images considered non-diagnostic due to significant motion blurring.

**Electronic supplementary material:**

The online version of this article (10.1007/s12350-020-02177-2) contains supplementary material, which is available to authorized users.

## Introduction

Unlike SPECT, in myocardial perfusion PET images are acquired throughout infusion of tracer and vasodilator stressing agents. This increases the likelihood of motion of the heart during the image acquisition introducing a varying degree of non-uniform blurring in the reconstructed images, hampering image interpretation and, in extreme cases, rendering the images either non-diagnostic or leading to incorrect interpretation.^1^–^4^ Motion remains an ever-present challenge in myocardial perfusion imaging. In rest-stress myocardial perfusion PET, the principal aim is to ensure that the static relative perfusion images preserve diagnostic image quality and it is these images that are arguably the most susceptible to this motion blurring, as a result of being averaged over several minutes.

We consider that there are three types of motion that can degrade static perfusion images: cardiac contraction; periodic motion, usually associated with regular breathing motion; and non-periodic motion. Non-periodic motion can be further divided into two sub-types: a gradual baseline drift of the heart position (sometimes referred to as “cardiac creep”) and irregular oscillation due to irregular breathing motion and movement as a result of discomfort arising from the stress agent side effects.^5^ At least one commercial software package is available which addresses the issue of cardiac contraction blurring to produce “Motion Frozen” static images.^6^,^7^ This only addresses cardiac contraction and so additional corrections are required to address the second and third types of heart motion discussed here. The impact of respiratory motion correction has been a focus of PET for many years—both in oncology and cardiology.^8^,^9^ External hardware options, such as optical systems or expandable belts, are available to estimate internal motion from external measurements from the patient and produce a finite number of respiratory gated images.^10^ These respiratory gates can be defined by either temporal (phase) or displacement (amplitude) methods, where image gates represent either equal time periods between end inspiration and end expiration or the position of an organ between end inspiration and end expiration, respectively. One method to utilize these respiratory gated images to perform motion correction in cardiac PET is to simply produce images of the myocardium from one respiratory gate or in the quiescent motion phase.^11^,^12^ However, this approach inevitably leads to inferior count statistics due to the division of acquired data into multiple gated images.^9^,^13^,^14^ Alternatively, it is possible to shift the position of the heart in each gate to align with one particular reference gate to retain the total number of counts.^15^,^16^ However, it has been suggested that this post-reconstruction alignment produces inferior image quality compared with inserting the motion information directly into the reconstruction process.^17^

While the use of external devices has been demonstrated to be effective, they require additional setup time and precise synchronization with the PET acquisition. They also exhibit additional technical challenges that may result in decreased robustness in a clinical setting.^18^ Consequently, several studies have investigated the use of data-driven respiratory motion tracking and correction in cardiac PET.^11^,^19^,^20^ However, these cases have evaluated data with either fluorine-18 FDG or nitrogen-13 ammonia but not rubidium-82, which has a substantially shorter half-life. The rapidly changing tracer kinetics of rubidium-82 may also provide an additional challenge^21^ that has not yet been exhaustedly addressed in the literature.

These aforementioned techniques to remove respiratory motion—either from external trigger or data-driven methods—are commonly based around the formation of a series of respiratory gated images of either fixed temporal or spatial width.^12^,^15^ While these methods may work when there is regular periodic breathing motion with a static baseline, they may not be the optimum technique for non-periodic motion in pharmacological stress-induced cardiac imaging.^13^,^16^ Two recent publications have highlighted the occurrence of non-periodic motion with a drift in baseline position in dynamic rubidium-82 reconstructions for calculation of myocardial blood flow (MBF).^22^,^23^

Current studies addressing motion in rubidium cardiac imaging have focused on the impact of inter-frame motion present in the dynamic reconstructions for MBF calculation.^22^,^24^–^26^ However in these cases, the error arises from incorrect time-activity curves due to segmentation errors. Simply applying frame-by-frame motion correction to the PET data by manual translation is usually sufficient to significantly reduce these errors and this functionality is available on some commercial software packages. Despite the value of motion correction in dynamic frames, there is very little work evaluating motion correction of static perfusion images. This is likely to be due to the difference in correction: dynamic motion correction is inter-frame translation, which is relatively straightforward, whereas motion correction of a static image is inherently intra-frame.

Frame-by-frame correction has been demonstrated in a single case study by Thompson et al. to be beneficial for static perfusion images.^4^ In this study, the authors reconstructed a non-diagnostic static perfusion image as a dynamic image of thirty 10-second frames. The individual frames were then aligned using rigid translations and summed to form a motion-corrected static image. Similar techniques have been recently presented from our institution.^2^ However, this was found to be impractical as reconstructing numerous multiple frames is labor and resource intensive and not considered practical for a center with high throughput. In addition, this frame-by-frame technique does not correct for motion that is high frequency to the degree that significant intra-frame motion still occurs in these short frames. The solution is to reconstruct a greater number of shorter frames to improve temporal sampling but this worsens image quality in each frame making visual alignment challenging and also heightens the burden on the reconstruction system.

The final aspect to consider in this study is the improvements from developments in scanner technology. Our institution uses a Biograph Vision 600 PET-CT scanner (Siemens Medical Solutions USA, Inc.) that employs Silicon Photomultipliers (SiPM) for signal detection that offer a significant increase of performance over systems that use traditional photomultipliers. The improved Time-of-Flight (TOF) performance, sensitivity and spatial resolution produces a notable improvement to image definition for rubidium-82 images in spite of the appreciable positron range of rubidium-82 (approximately 5 mm in soft tissue).^27^ Having performed nearly 2000 rest-stress rubidium scans on the Biograph Vision, the degrading impact of motion on the static perfusion images is more apparent and hence the correction for this becomes ever more desired.

In this work, we present preliminary findings from a prototype Data-Driven Motion Correction (DDMC) algorithm that has been specifically designed for cardiac PET applications. The algorithm acts on listmode data prior to reconstruction and hence eliminating the need to reconstruct and align multiple short images. We leverage the improved TOF performance, sensitivity, and spatial resolution possible with a modern SiPM PET system to perform motion correction at a temporal resolution that has not previously been possible. The algorithm has been applied to phantom and patient studies.

## Methods

### Data-Driven Motion Correction

**Figure 1:**
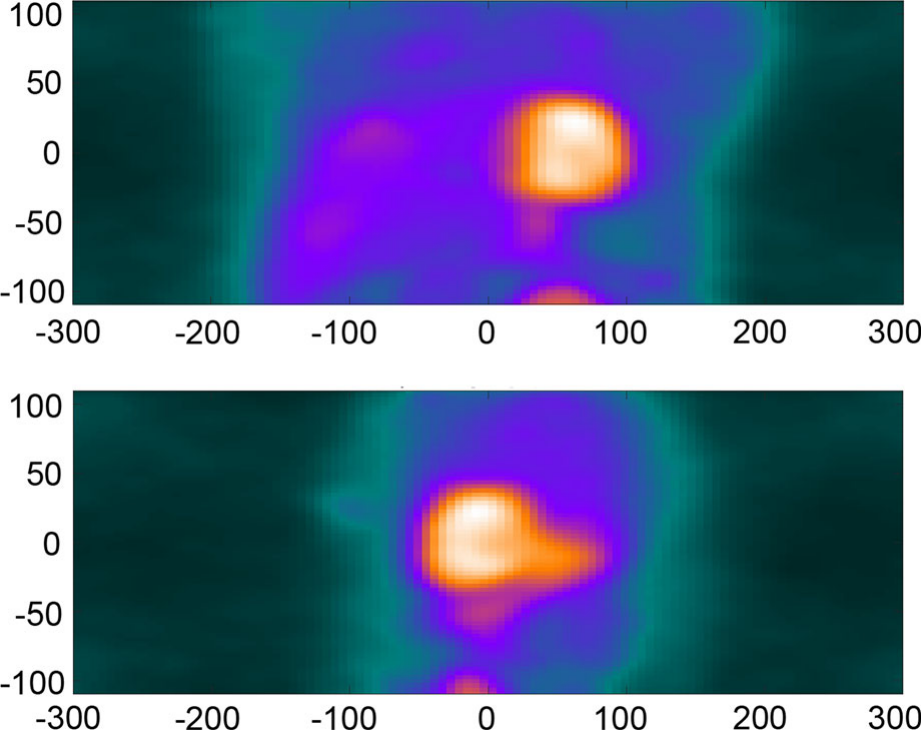
Maximum intensity projections of a Direct Volume Histogram (DVH) frame created from binning the position of positron annihilation events using TOF information shown from the anterior coronal view (top) and the left-lateral view (bottom). The DVH frame represents one second of coincidence events and was obtained at 180 seconds after the start of the image acquisition. Axis values represent distance in mm

The DDMC algorithm implemented in this work tracks the position of the heart using coincidence information extracted from the PET raw listmode data. Instead of binning lines of response recorded in the listmode data into sinograms for reconstruction as is performed traditionally, the position of positron annihilation events is binned directly into a volume, referred to as a Direct Volume Histogram (DVH), according to the TOF difference along the line of response.^2^ The dimensions in the *x*, *y*, and *z* direction of the DVH bins are 8, 8, and 6 mm, respectively. The TOF resolution of the Biograph Vision is 214 ps resulting in a spatial localization precision of approximately 3 cm for the positron annihilation. This produces a relatively low spatial resolution image that is not sufficient for clinical interpretation but is adequate to locate and track the rigid translation motion of the heart. These approximations allow for a motion measurement achievable in a clinical timeframe, available within seconds of the scan completion. No correction for attenuation, scatter, or detector normalization is performed in the creation of the DVH. Only an axial sensitivity correction is applied to equalize the intensity of all planes. One DVH frame is created for every second of data collected and hence, for the clinical protocol here of 5 minutes, 300 DVH frames are created. The use of half-second DVH frames was evaluated but it was found to result in increased noise and inferior performance. Figure 1 shows an example of a 1-second DVH frame.

Unlike other data-driven motion tracking techniques, the DDMC algorithm specifically locates and tracks the rigid translation of the heart over the course of the acquisition. The heart is automatically located within the DVH volume by searching for a relatively high-intensity area of activity that is the approximate size and typical location in the DVH volume. This is performed at frame 180 which, as shown by Figure 1, allows clearance of extra-cardiac activity, with the myocardium being well perfused and is used as reference image for other DVH frames.

The *x*, *y,* and *z* parameter space is searched to return the position that maximizes the normalized cross-correlation coefficient with a constrained region around the heart for the particular time point compared with the reference frame. This is performed for all DVH frames from 120 to 300 seconds (the temporal framing for the static perfusion images) and produces a motion vector in all 3 dimensions over the time of the perfusion scan. This *z* component of the motion vector is subsampled and used as an axial shifting during the sinogram rebinning process to produce a “motion-corrected” sinogram at its inherent resolution of 1.6 mm. In the current implementation, only the axial component (*z*-axis) of the translation is incorporated into the correction, and it is incorporated without adjustments for normalization, scatter, or randoms correction. These additional corrections along with integration of *x* and *y* motion will all be explored in the future but are expected to be minor effects based on the results observed to date.

A non-attenuation-corrected (NAC) image is reconstructed in the standard clinical protocol for the purposes of checking registration between the PET and the CT. This same registration and timing is used to register the motion-frozen PET image to the CT for attenuation correction alignment so that any additional shift required to register the PET to CT is incorporated into the reconstruction. Other organs in the PET field of view are likely to exhibit motion that is different to the heart. Therefore, it is important to note that since the motion of the heart is used in correcting the entire PET volume, all other areas in the volume may have attenuation artifacts and should not be used for any quantitative ancillary findings.

### Phantom Data

PET-CT acquisitions of an anthropomorphic torso phantom with a cardiac insert (Data Spectrum, Durham, NC, United States) were used to provide a ground truth for the initial assessment of the DDMC algorithm. The phantom was filled with a total of 50 MBq of fluorine-18 FDG such that the cardiac insert had an activity concentration of 10 times that of the background activity. A solid defect was placed in the cardiac insert and used to assess the recovery of an apparent localized perfusion defect. The scanner used was a Siemens Biograph Vision 600 SiPM PET-CT system.

The phantom was placed on a low friction nylon sheet on the imaging couch. A CT was performed followed by two listmode acquisitions, each lasting three minutes. The first acquisition was with the phantom stationary and the second was with the phantom being manually pulled to-and-fro in the axial direction following a random motion pattern. This stationary acquisition was intended to serve two purposes: firstly as a ground truth comparison for DDMC images of the moving phantom and secondly as a negative control test to determine that applying DDMC to a stationary object did not produce any unforeseen results.

For each image, two sets of sinograms were produced from the full 3 minutes of listmode data: the original data with no motion correction (NMC) and a second incorporating the DDMC. From the sinograms, static images were reconstructed with a 3D OSEM iterative reconstruction algorithm incorporating both TOF and point spread function modeling with 4 iterations, 5 subsets, and a 6.0 mm FWHM Gaussian post-filter. The matrix was 220 × 220 with a zoom of 2.0 and 159 transaxial slices giving voxel dimensions 1.6 × 1.6 × 1.6 mm^3^.

The cardiac insert was orientated to the cardiac axis and assessed visually in QPET (Cedars Sinai, Los Angeles, CA, United States). Quantitative analysis of the defect severity was performed using the total perfusion deficit (TPD) from the software. Profiles were drawn along the axial direction through the cardiac insert without any cardiac orientation to avoid voxel interpolation effects and the FWHM of the wall thickness was determined.

## Clinical Data

### Patient Selection and Image Acquisition

46 images (29 stress and 17 rest) from 36 patients who underwent clinically indicated rubidium-82 perfusion scans were retrospectively selected based upon a perception of motion blurring in the static perfusion images. The patient demographics from these cases are given in Table 1. A Sharpiro–Wilk test for normality of all continuous variables was performed and all data were found to be normally distributed. All data were fully anonymized prior to analysis.

**Table 1:** Patient demographics for this study

Total number patients	36	
Gender	28M; 8F	
Age (years)	60 ± 11	
Weight (kg)	92.3 ± 22.6	
Height (m)	1.70 ± 0.08	
BMI (kg/m^2^)	33.7 ± 6.9	
Resting systolic BP	126 ± 21	
Resting heart rate	71 ± 13	
Number of stress images	29	
Stress agent	Adenosine	24
	Regadenoson	5

All patients were required to abstain from caffeine for 12 hours prior to imaging. A single low-dose CT scan (120 kV; 11 mAs quality reference with CAREDose modulation) was acquired during free-breathing for attenuation correction purposes. This was followed by a rest-stress protocol with 740 MBq (20 mCi) of rubidium-82 administered for both rest and stress. A 5-minute listmode acquisition was started as the rubidium was administered. Pharmacological stress was used in all patients, with the choice of agent shown in Table 1. The adenosine protocol consisted of a 4.5-minute infusion of 140 µg·min^−1^·kg^−1^ with the rubidium infusion commencing 2 minutes after the start of the adenosine infusion. The regadenoson protocol consisted of a fixed 400 µg dose in 5 mL that was administered slowly over 20 seconds. This was followed by a 5 mL saline flush administered over 10 seconds. The rubidium infusion was started 60 seconds after the saline flush was given.

### Motion Characterization

There are two components to the motion of the heart that degrades image quality: the general oscillation about a baseline position, and whether there is any drift in this baseline position over the course of the image acquisition. Preliminary work with DDMC showed that sustained oscillation of greater than ± 6 mm about a baseline position would give rise to appreciable degradation of image quality.^2^ Any cases here exhibiting such oscillation greater than ± 6 mm were classified as exhibiting periodic or irregular oscillation depending on the nature of motion; cases with less than ± 6 mm were classified as negligible oscillation. In addition, the presence of any baseline drift was assessed.

To compare the magnitude of motion along the three orthogonal directions, the mean position of the heart across the duration of the static image framing was determined and then the mean of all displacement vectors in the *x* (left-right transaxial), *y* (anterior-posterior transaxial), and *z* (axial) directions were determined for each image.

### Image Reconstruction

An NAC PET image was reconstructed from 120 to 150 seconds into the acquisition for registration of the PET data to the CT, which is part of our standard clinical protocol and allows this NAC image to be reconstructed and used for registration adjustment before the acquisition has completed. For the attenuation-corrected reconstructions, the listmode data were framed into sinograms over the time range of 120 to 300 seconds after the start of the scan. As with the phantom data, two sets of sinograms were produced for each patient image: an NMC sinogram and a DDMC sinogram. In both cases, any adjustment translation in PET to CT registration obtained from the NAC image was applied.

From these sinograms, static perfusion images were reconstructed with a 3D OSEM iterative reconstruction algorithm incorporating both TOF and point spread function modeling with 4 iterations, 5 subsets, and a 6.0 mm FWHM Gaussian post-filter. The matrix was 220 × 220 with a zoom of 2.0 and 159 transaxial slices giving voxel dimensions of 1.6 × 1.6 × 1.6 mm^3^.

### Image Interpretation

The images were blinded, randomized, and then presented as image pairs (one NMC and one DDMC) to a physician experienced in nuclear cardiology PET reporting. Each dataset was scored on image quality as follows: non-diagnostic (score 0), adequate (score 1), and good (score 2); and perceived motion: none (score 0), mild (score 1), moderate (score 2), and severe (score 3).

## Results

### Phantom Data

**Figure 2:**
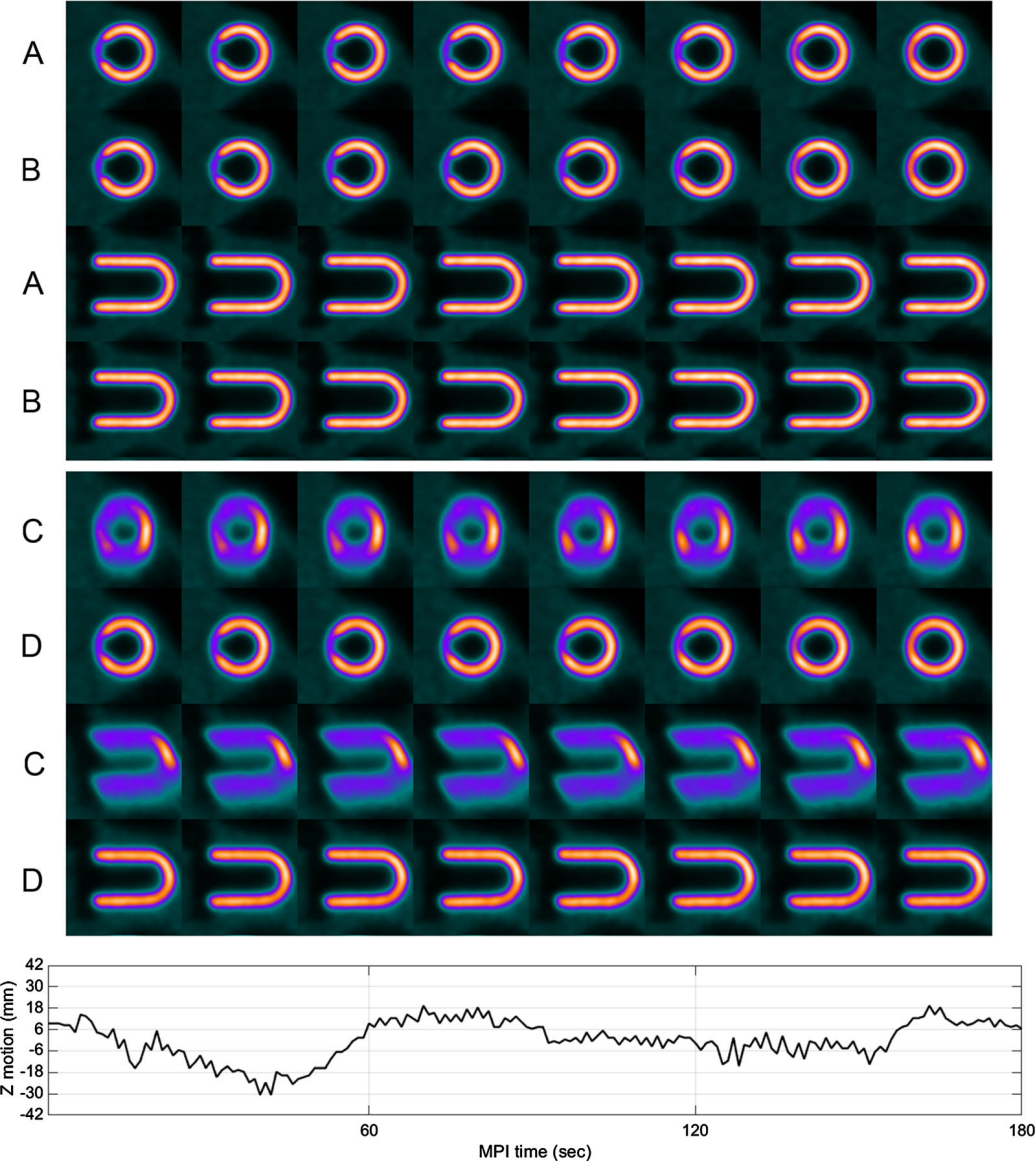
Short axis and vertical axis slices of the cardiac phantom acquired while stationary with no motion correction (**A**) and with the DDMC applied (**B**); phantom acquired while moving with no motion correction (**C**) and with the DDMC applied (**D**). The motion trace at the bottom was extracted from the DDMC motion tracking and shows the displacement of − 30 mm to + 18 mm in the axial direction where the reference position is the average axial position over the acquisition

**Figure 3:**
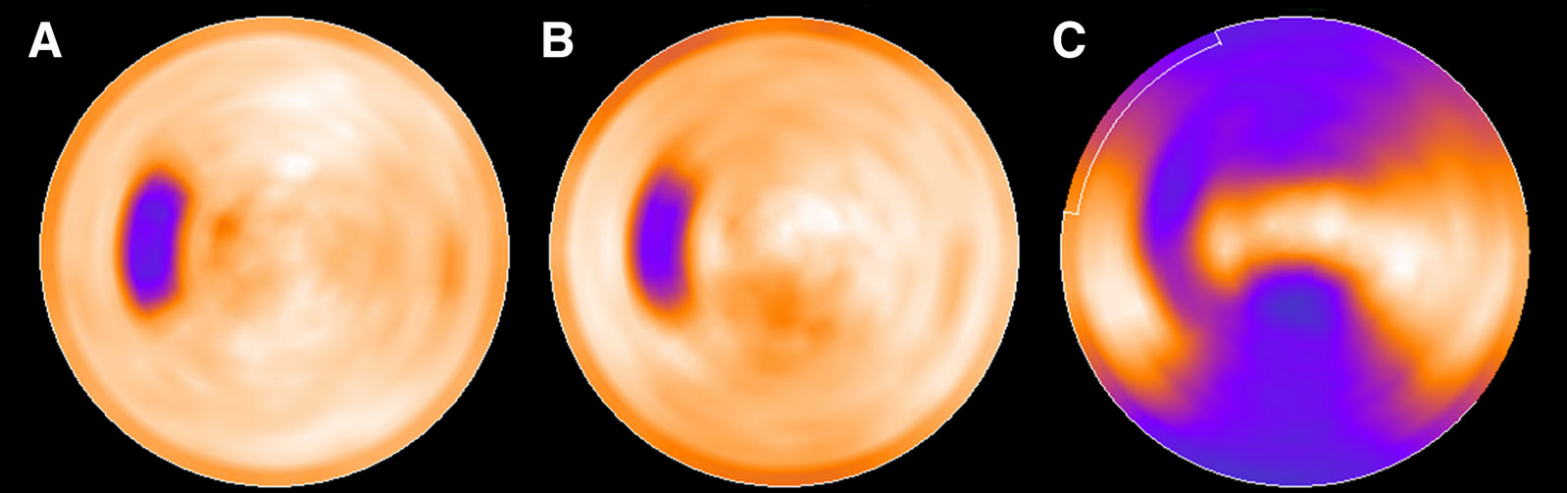
Polar maps of the cardiac phantom showing defect visualization. Images from the stationary phantom without correction (**A**), the moving phantom with DDMC applied (**B**), and the moving phantom without correction (**C**)

**Figure 4:**
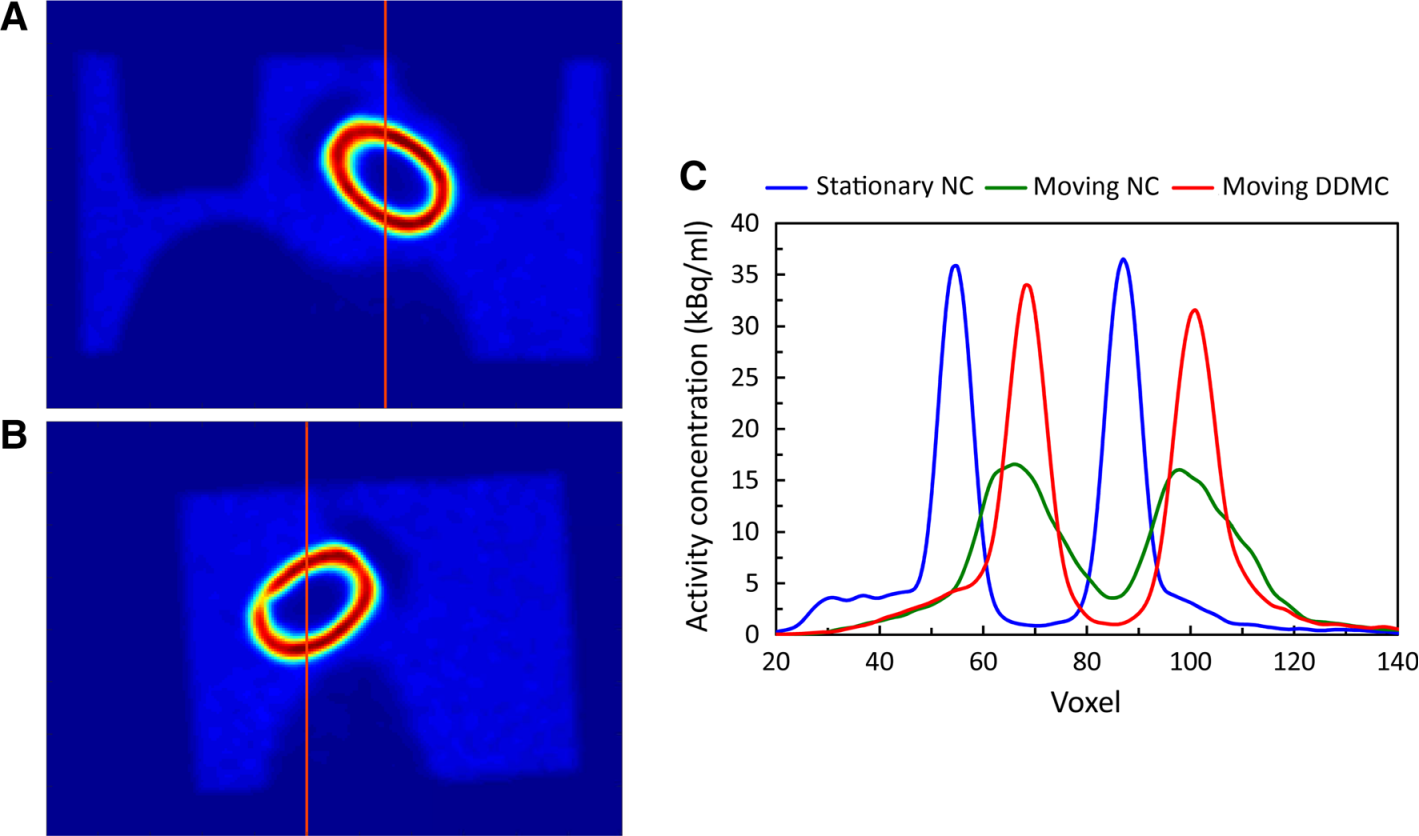
Coronal (**A**) and sagittal (**B**) images of the stationary phantom used to illustrate the position of the axial profiles (**C**) that were defined through the stationary and moving phantom without DDMC. The axial offset in the stationary NMC and moving DDMC curves arises from the positional reference for the DDMC being in the center of the motion trace

The original and DDMC phantom images from the stationary and moving image acquisitions are shown in Figure 2 together with the motion trace obtained from the DDMC algorithm. Negative control evaluation of performing DDMC on the stationary phantom data resulted in images identical to the NMC images being produced. For the moving phantom, there was clear improvement with DDMC. The TPD was 10% for both the NMC and DDMC images of the stationary phantom and for the DDMC images of the moving phantom. The TPD for the NMC images of the moving phantom was 42%. The polar plots for the stationary and moving phantoms are shown in Figure 3. The FWHM of the cardiac insert axial profiles shown in Figure 4 was 11.8 mm and 13.1 mm for the stationary and moving phantom with DDMC, respectively.

### Clinical Data

Table 2 shows the categorization of the types of motion and illustrates the diverse types of motion observed. As can be seen, the 42 cases considered to exhibit oscillation are divided equally into periodic and irregular.

**Table 2:** Categorization of the types of motion observed in this study

	Negligible baseline drift	Baseline drift	Total
Periodic oscillation	16	5	21
Irregular oscillation	10	11	21
Negligible oscillation		4	4
Total	26	20	46

**Figure 5:**
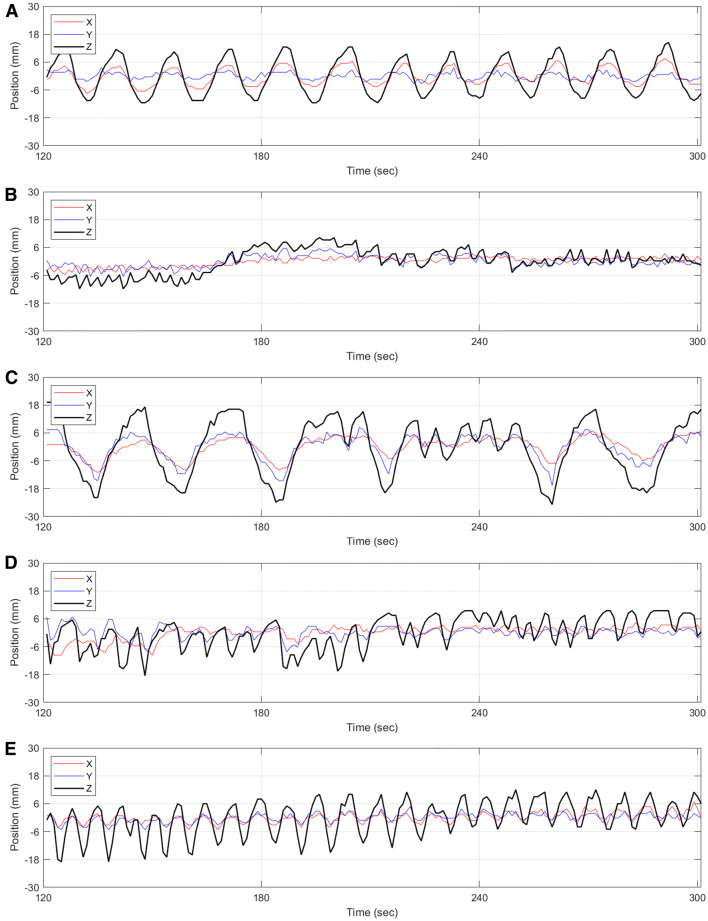
Motion traces from five patients extracted from the DDMC tracking over the 3-minute period of the acquisition used to produce the static perfusion images. These plots show examples of the types of motion and are categorized as **A** periodic oscillation with negligible baseline drift, **B** negligible oscillation with baseline drift, **C** irregular oscillation with negligible baseline drift, **D** irregular oscillation with baseline drift and **E** periodic oscillation with baseline drift. While DDMC is currently only performed along the axial direction (*z*-axis), the motion tracking of the *x* and *y*-axis is also performed and shown here

**Figure 6:**
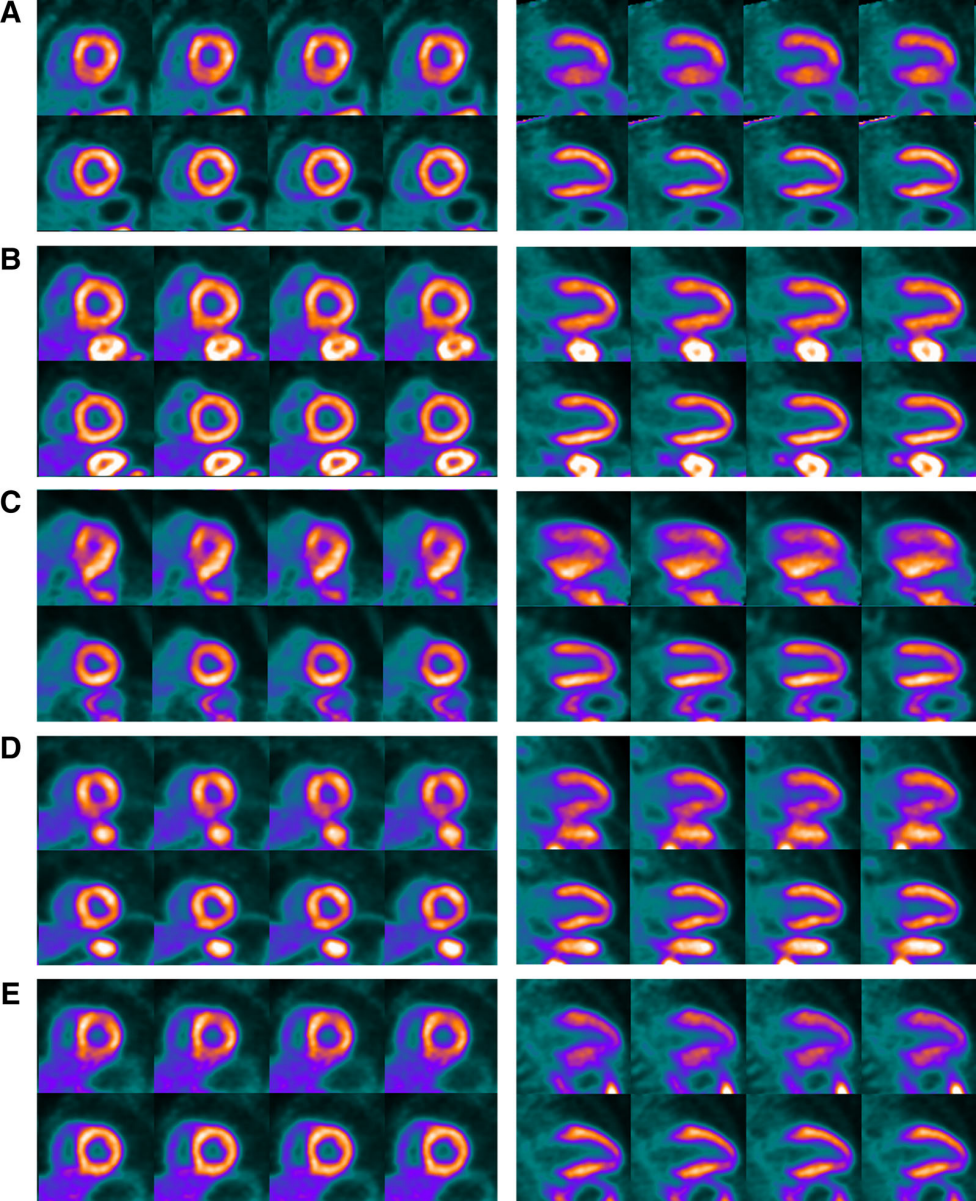
Short axis and vertical slices for the five cases corresponding to the motion traces shown in Figure 5. In each case, the NMC images are shown above the DDMC images

Figure 5 shows motion traces from five patients to illustrate the varieties of motion characteristics that were observed in this study. It is useful to note that the case in Figure 5C exhibited the greatest magnitude of motion for the clinical cohort and this was less than the magnitude of motion that was produced for the phantom study shown in the motion trace of Figure 2. As stated, the current implementation of the DDMC only corrects data in the *z* (axial) direction but the motion tracking element does extract the displacement in the *x* (left-right transaxial), *y* (anterior-posterior transaxial) directions. The accompanying NMC and DDMC perfusion images for these five cases are shown in Figure 6.

**Figure 7:**
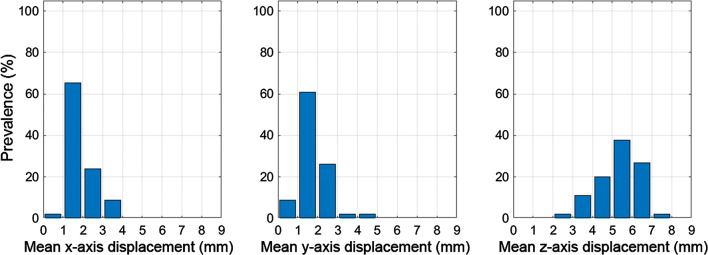
Histograms of the mean displacement in the *x*, *y*, and *z* directions. It should be noted that the number of images in each bin has been normalized as a percentage of the total number of images (46)

Figure 7 shows histograms of the mean displacement in the x, y, and z directions. It can be seen that over 60% of images exhibit a mean displacement of less than 2 mm, which is approximately one voxel or less, in either x and y direction, while there is considerably greater displacement along the *z*-axis.

**Figure 8:**
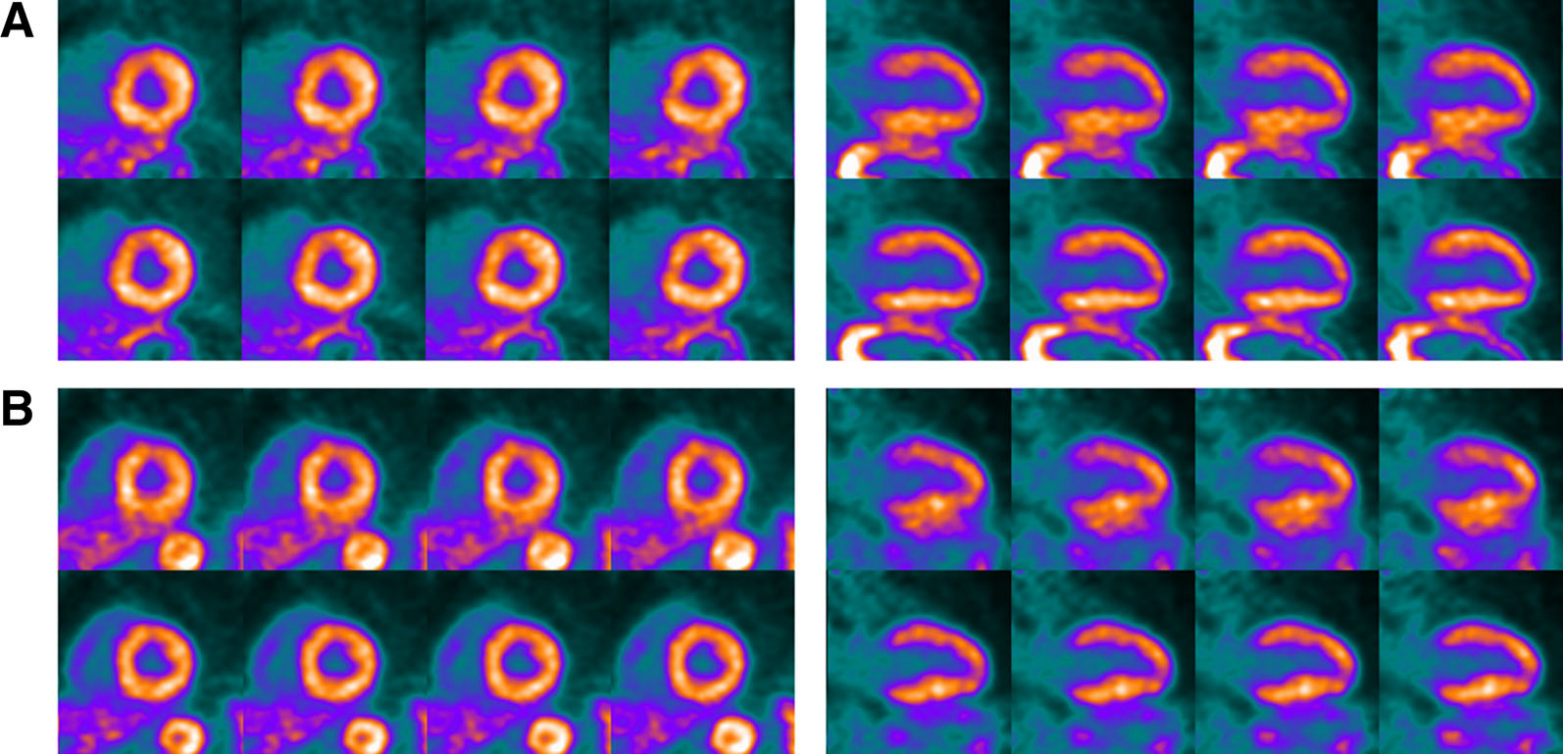
Images from two patients with high BMI, case *A* = 42 kg/m^2^; case *B* = 37 kg/m^2^, where the DDMC images (lower set of images for each case) were considered to have mild motion. The four sole sets of images considered to have mild motion were from these two patients

For the 46 rubidium images, quality was rated as follows: NMC images: 10 non-diagnostic, 31 adequate, and 5 good; DDMC images: 0 non-diagnostic, 6 adequate, and 40 good. Perceived motion was rated as follows: NMC images: 4 no motion, 24 mild, 11 moderate, and 7 severe; DDMC images: 42 no motion and 4 mild. Tables 3 and 4 show the frequency of differences in scores for image quality and perceived motion. As can be seen, no DDMC images were considered to have inferior quality compared with NMC images and, likewise, no DDMC images were considered to have greater perceived motion compared with NMC images. The four cases that were rated as still having mild motion in the DDMC images were from two patients. The two patients had high BMI (37 and 42 kg/m^2^) and perfusion images were seen to be noisy and so this was considered to be the reasoning for this rating. Rest images are shown from each of these patients in Figure 8.

**Table 3:** Frequency of images with various differences of image quality scores

Difference in image quality (DDMC-NMC)	Number of images
2	8
1	29
0	9
− 1	0
− 2	0

A positive difference indicates improved quality in the DDMC images

**Table 4:** Frequency of images with various differences of perceived motion scores

Difference in perceived motion (DDMC-NMC)	Number of images
3	0
2	0
1	0
0	4
− 1	24
− 2	13
− 3	5

A negative difference indicates reduced perceived motion in the DDMC images

## Discussion

In this work, we have presented a preliminary evaluation of a prototype DDMC algorithm that incorporates a high temporal resolution tracking, which has been created specifically for cardiac PET. The implementation of the DDMC algorithm does not divide data into a discrete number of respiratory gates and instead is designed to automatically detect and track the displacement of heart and align to a predefined reference location. This design is intended to accommodate non-periodic motion of the heart under stress conditions and hence accounting for irregular oscillation as well as a shifting in the baseline position of the heart. To the best of our knowledge, this is the first study of its type to evaluate data-driven motion correction for rest-stress rubidium cardiac PET based on positional tracking of the heart where no coincidence events are discarded.

The phantom data, intended to give a ground truth, showed the following: DDMC had no impact on the images when applied to the stationary phantom images; the myocardial wall thickness was comparable in the motion-corrected moving phantom images compared with the stationary phantom and the defect was recovered well, both visually and quantitatively, when compared with the stationary images.

We observed several different types of heart motion during the clinical rest-stress perfusion images. Some of the cases in this study exhibited periodic oscillation of the heart position with negligible baseline drift. It is possible to hypothesize that these cases may be corrected adequately by conventional respiratory motion correction techniques. However, as shown by Table 2, only 16 of the 46 cases were classified as such with the remaining cases either exhibiting baseline drift or irregular oscillation, illustrating the complex and diverse forms of motion experienced in cardiac PET. This highlights the need for a flexible motion correction strategy to accommodate this. In a study using 12 patients, Martinez–Moller also reported that half of the patients exhibited considerable baseline drift. The study also stated that amplitude-based respiratory gating did not work effectively in these cases.^16^

A recent study of data-driven respiratory gating for rubidium imaging by Manwell et al used external fiducial radioactive sodium-22 markers rather than the myocardium to produce six separate images at different phases of the breathing cycle.^11^ The aim of their study was to benchmark the use of these external markers against and external system rather than using the rubidium data.

Lassen et al employed a similar strategy to our work of detecting and tracking the position of heart in sinogram space to perform data-driven motion correction.^20^ At an intermediate stage of their technique, four amplitude-based respiratory gates were produced from the motion tracking which were then registered to the end expiration gate. Their study was performed on seven patients undergoing dual-tracer N-13 ammonia and F-18 FDG myocardial viability studies acquired with the patients in a resting state.

The shifting of the heart in the axial direction to a reference location using a rigid translation has been previously demonstrated.^15^ However, a small feasibility study by Matrinez-Moller et al showed varying degrees of craniocaudal motion in the different myocardial walls^16^ suggesting that deformable transformation was required. Their study reported mean ± SD displacement of 4.0 ± 1.5 and 5.5 ± 2.2 mm for the anterior and inferior wall, respectively. The DVH volumes used here do not have sufficient spatial resolution to measure any elastic deformation of the heart due to motion; however, the displacement observed in our study is considerably greater and we feel that any differences in wall displacement would be negligible compared with this overall magnitude of motion. In addition, the 1-second temporal sampling would not be sufficient to assess any deformation of the heart due to contractile motion.

## Limitations

In this implementation of the DDMC algorithm, motion correction is only performed along the axial direction (*z*-axis). The data in Figure 7 demonstrate that motion is predominantly along the z-axis, which is supported by other studies.^13^,^15^,^18^ Hence, we feel that the value for correcting only *z*-axis motion is demonstrated here.

In this currently prototype version of the algorithm, the axial normalization and crystal normalization and sensitivity are not addressed strictly during the motion correction. It is difficult to quantify these effects but the phantom study implies that degradation is negligible and the magnitude of displacement is considerably greater than the displacement observed in any of the clinical images. The axial field of view of the Biograph Vision here is 26 cm and hence, while the observed motion is of sufficient magnitude to cause image blurring, motion of the heart is still small compared with the portion of the axial FOV that it is moving over. Therefore, it is anticipated that any influence from varying axial sensitivity is expected to be very small. However, as mentioned these corrections will be the focus of future work.

The current implementation of the algorithm is only applicable to static perfusion images. This is due to current limitations of the process in which the motion-corrected sinograms are generated from the listmode data. By inserting motion information into the listmode, as performed by previous studies,^15^,^20^ it would be possible to reconstruct DDMC ECG-gated and dynamic images for wall motion and myocardial blood flow, respectively. Work is ongoing in our institution to insert these displacement tags into the listmode file. An additional benefit of ECG-gated images with DDMC would be in the use of commercial software to produce motion-frozen static images where cardiac contraction blurring is also eliminated.

The patients included in this study were selected based on cases where substantial motion blur was perceived to affect image interpretation by an experienced nuclear medicine physician. It does not demonstrate the prevalence of motion in routine rubidium-82 myocardial perfusion PET. Based on previous work from our institution looking at dynamic studies, severe motion occurs in around 4% of cases.^5^ It may be the case that the portion of images with subtle motion blur is far greater and hence the true value of this correction remains to be seen. We plan to evaluate a large number of non-selective consecutive cases which intends to show the prevalence and severity of motion in routine rubidium myocardial perfusion PET.

There is currently no ground truth in the clinical data to show any improvement in diagnostic accuracy and ability to visualize reductions in regional perfusion more confidently. However, the aim of this preliminary study was to demonstrate the ability of the DDMC algorithm to transform non-diagnostic images into diagnostic quality images, which we feel has been clearly shown. The phantom data demonstrated no adverse effects of DDMC when compared with the stationary phantom images. A follow-on study is being planned to compare with clinical patient follow-up and correlation with other cardiac imaging to determine the diagnostic accuracy of the DDMC images.

## New Knowledge Gained

Substantial motion of the heart has been observed during rubidium rest-stress myocardial perfusion imaging with the motion patterns observed being highly variable. Data-driven motion correction using positional tracking of the myocardium is possible for rubidium cardiac PET scans, despite the short half-life of this tracer. Current state-of-the-art TOF performance has allowed for a novel approach to produce high temporal resolution images allowing accurate positional motion tracking.

## Conclusion

A new data-driven motion correction algorithm, designed specifically for cardiac imaging, has been developed and evaluated. Phantom data with motion show that the algorithm gives near-perfect recovery of myocardial wall visualization and defect quantification. The algorithm was applied to 46 clinical rubidium images exhibiting evidence of motion blurring where 10 images were considered non-diagnostic. All corrected images were interpretable. Work is ongoing to expand the application of this technique to ECG-gated and dynamic reconstructions.

## Electronic supplementary material

Below is the link to the electronic supplementary material.

Electronic supplementary material 1 (PPTX 818 kb)

## Abbreviations

DDMC: Data-driven motion correction
DVH: Direct volume histogram
NAC: Non-attenuation corrected
NMC: Non-motion corrected
PET: Positron emission tomography
TOF: Time-of-flight
TPD: Total perfusion deficit
