# Supplementary material for: A preliminary evaluation of a high temporal resolution data-driven motion correction algorithm for rubidium-82 on a SiPM PET-CT system
Source: J Nucl Cardiol. 2020 May 21;29(1):56–68. doi: 10.1007/s12350-020-02177-2 (PMC8873161; doi:10.1007/s12350-020-02177-2)
Supplement: Supplementary file 1 — Electronic supplementary material 1 (PPTX 818 kb) [file 12350_2020_2177_MOESM1_ESM.pptx]

## Slide 1
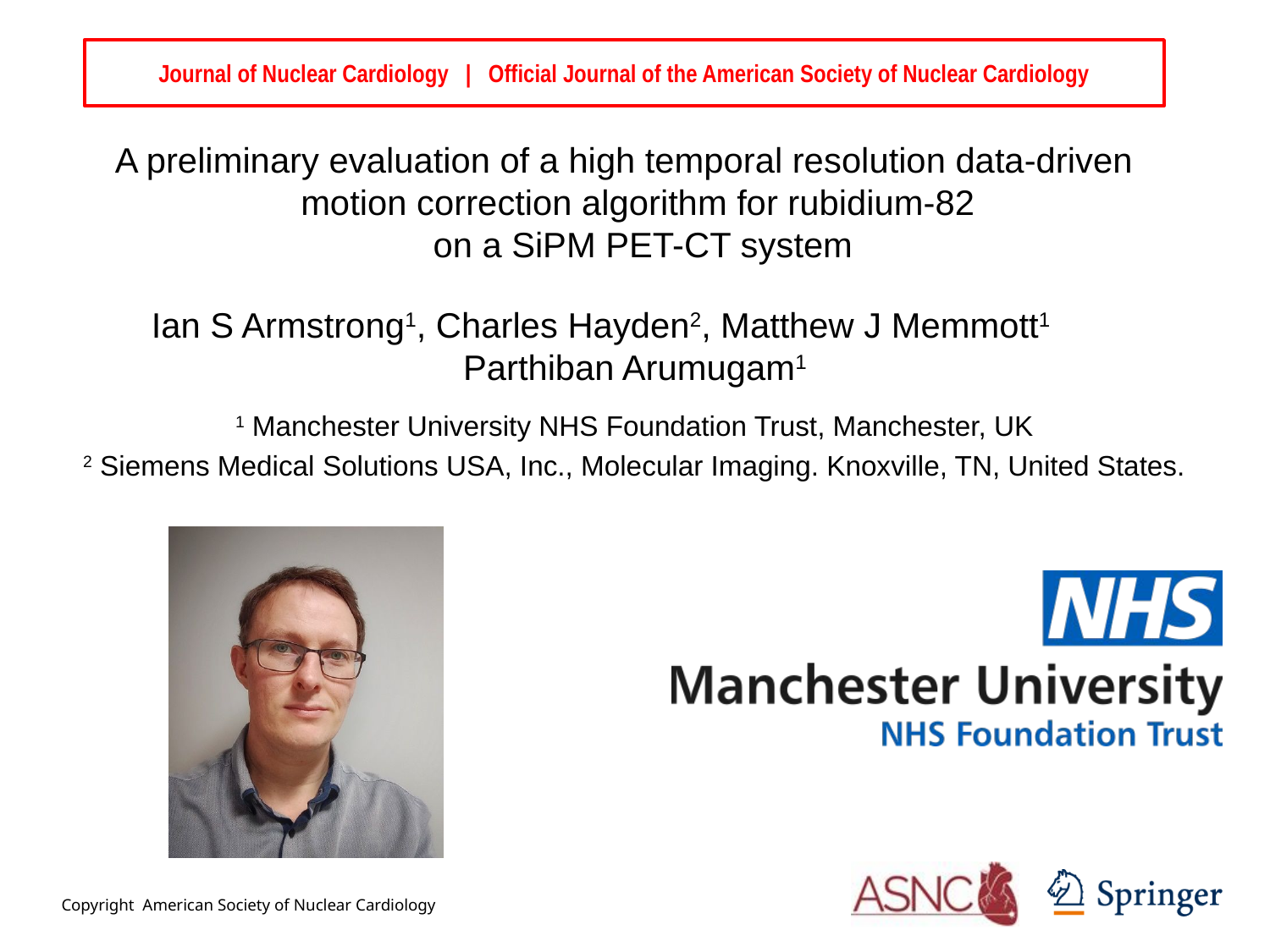

Journal of Nuclear Cardiology | Official Journal of the American Society of Nuclear Cardiology
# A preliminary evaluation of a high temporal resolution data-driven motion correction algorithm for rubidium-82 on a SiPM PET-CT system
Ian S Armstrong1, Charles Hayden2, Matthew J Memmott1 Parthiban Arumugam1
1 Manchester University NHS Foundation Trust, Manchester, UK
2 Siemens Medical Solutions USA, Inc., Molecular Imaging. Knoxville, TN, United States.
Copyright American Society of Nuclear Cardiology

## Slide 2
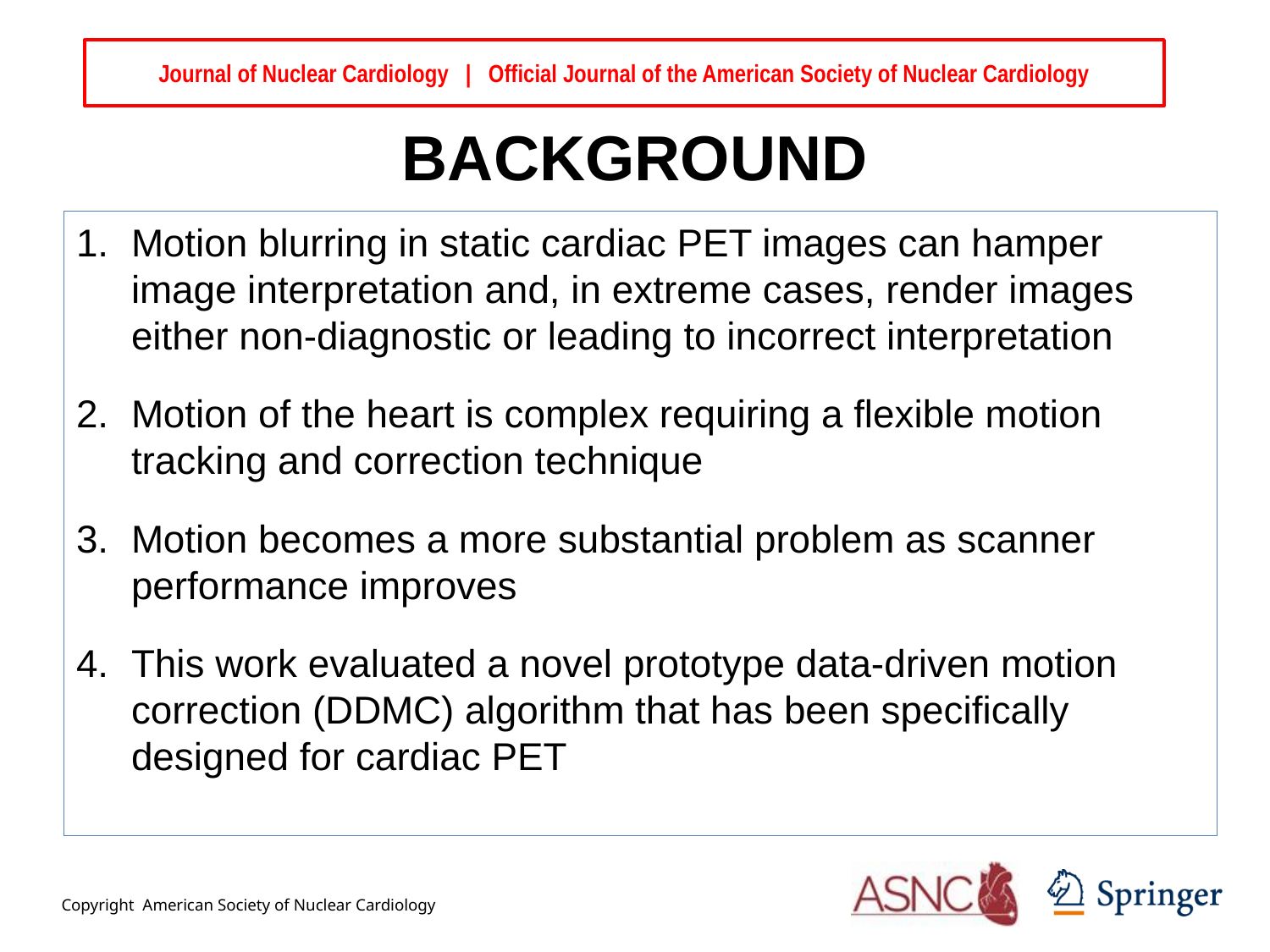

Journal of Nuclear Cardiology | Official Journal of the American Society of Nuclear Cardiology
# BACKGROUND
Motion blurring in static cardiac PET images can hamper image interpretation and, in extreme cases, render images either non-diagnostic or leading to incorrect interpretation
Motion of the heart is complex requiring a flexible motion tracking and correction technique
Motion becomes a more substantial problem as scanner performance improves
This work evaluated a novel prototype data-driven motion correction (DDMC) algorithm that has been specifically designed for cardiac PET
Copyright American Society of Nuclear Cardiology

## Slide 3
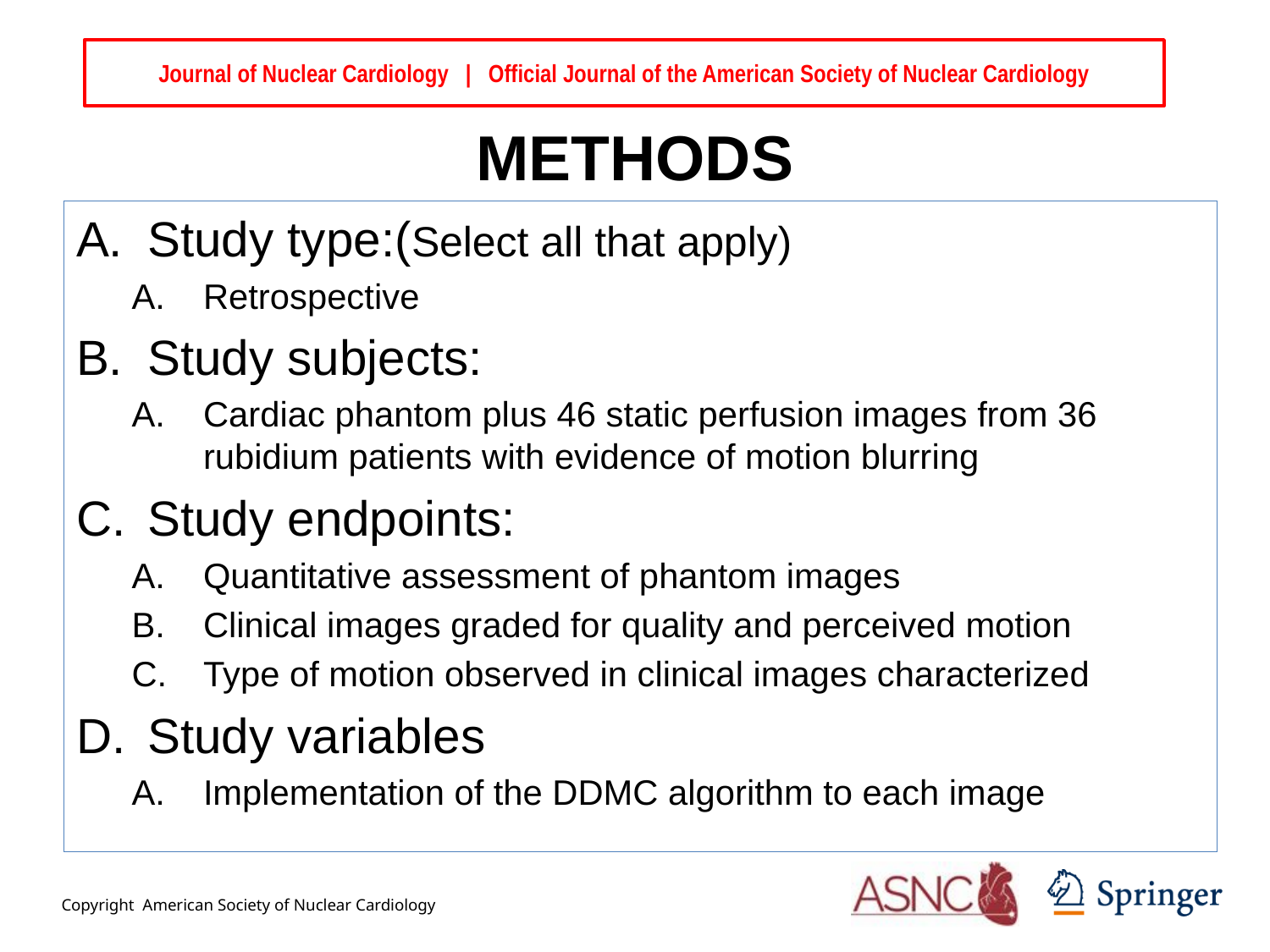

Journal of Nuclear Cardiology | Official Journal of the American Society of Nuclear Cardiology
# METHODS
Study type:(Select all that apply)
Retrospective
Study subjects:
Cardiac phantom plus 46 static perfusion images from 36 rubidium patients with evidence of motion blurring
Study endpoints:
Quantitative assessment of phantom images
Clinical images graded for quality and perceived motion
Type of motion observed in clinical images characterized
Study variables
Implementation of the DDMC algorithm to each image
Copyright American Society of Nuclear Cardiology

## Slide 4
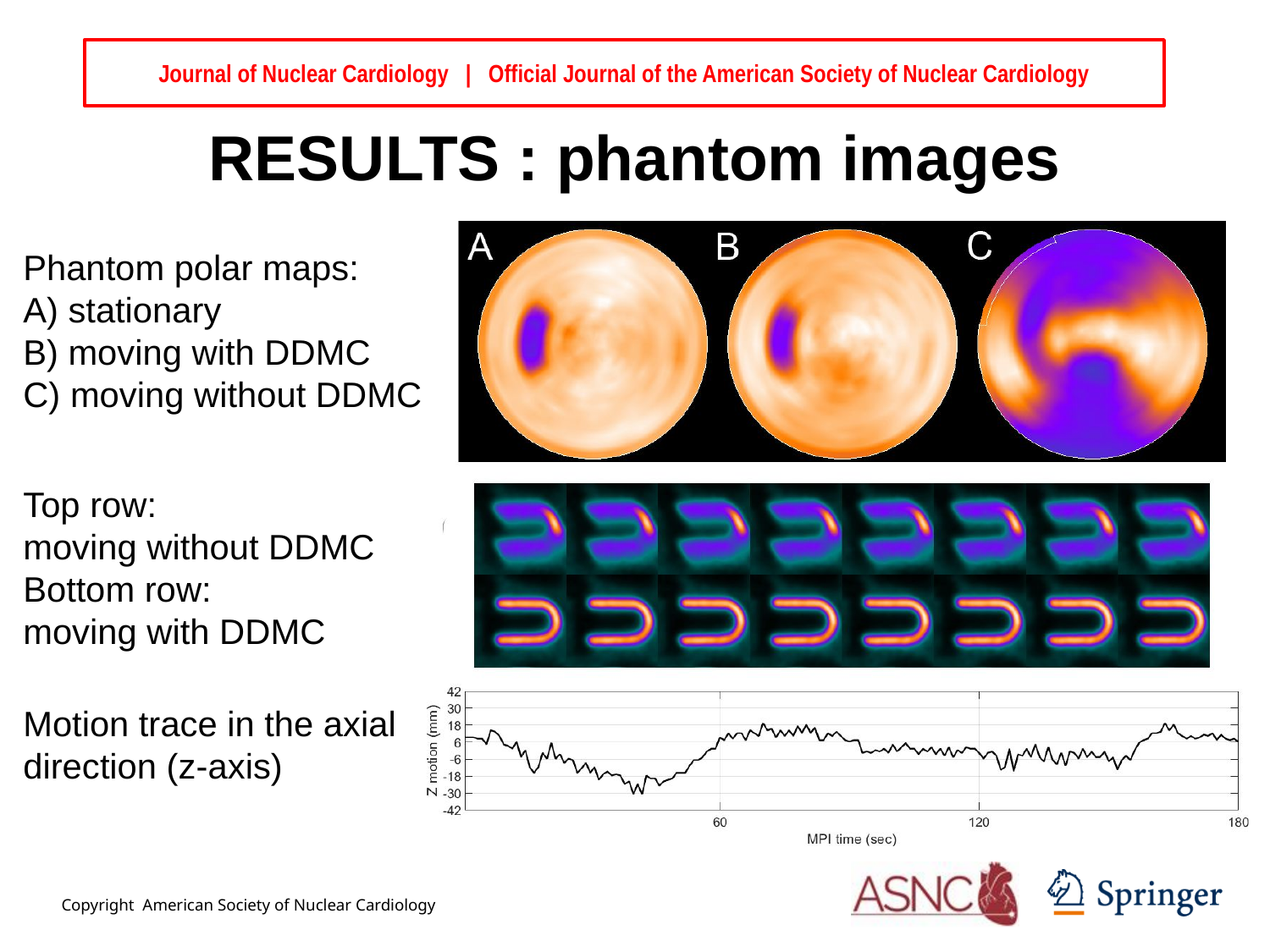

Journal of Nuclear Cardiology | Official Journal of the American Society of Nuclear Cardiology
# RESULTS : phantom images
Phantom polar maps:
A) stationary
B) moving with DDMC
C) moving without DDMC
Top row:
moving without DDMC
Bottom row:
moving with DDMC
Motion trace in the axial direction (z-axis)
Copyright American Society of Nuclear Cardiology

## Slide 5
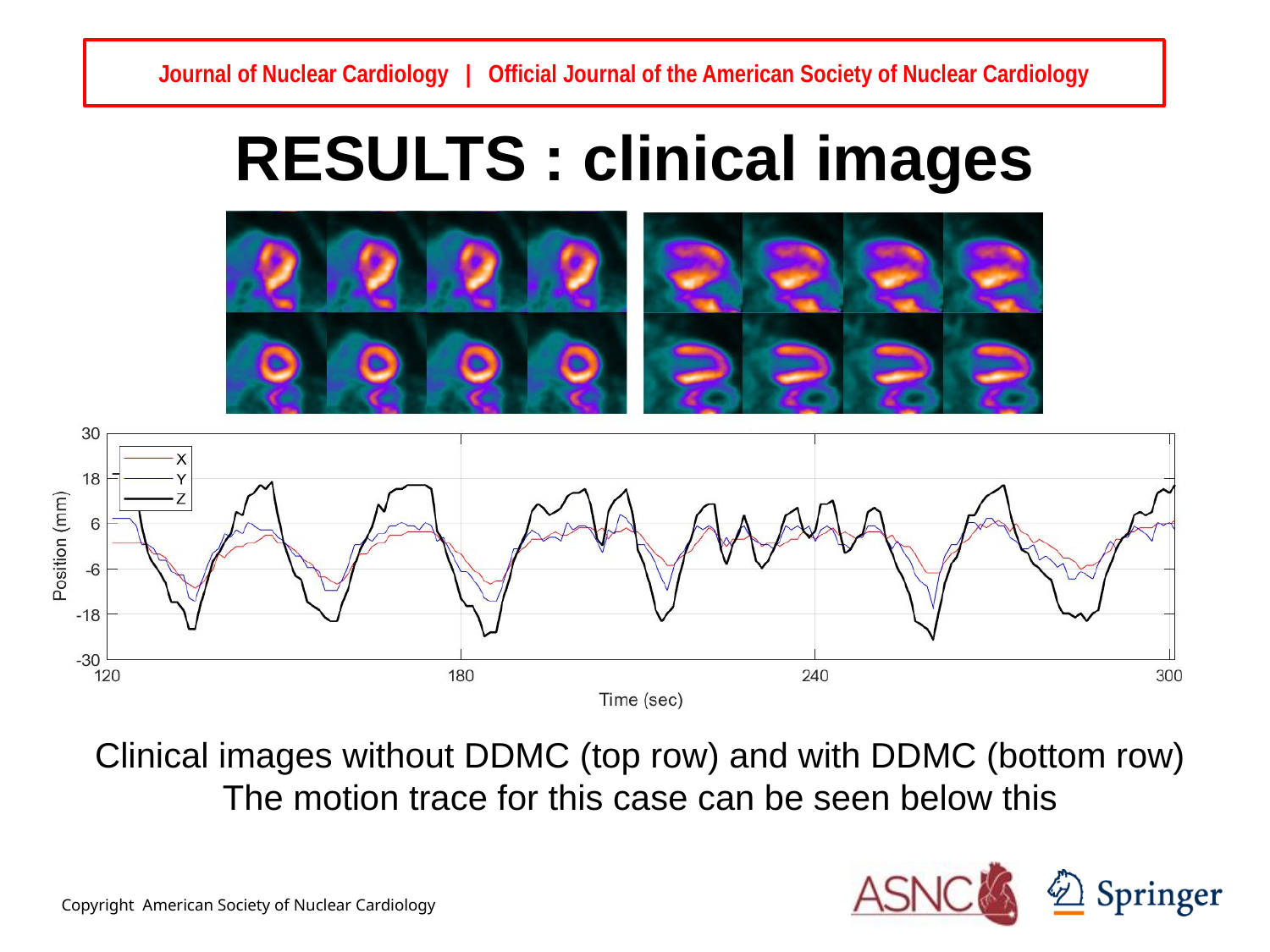

Journal of Nuclear Cardiology | Official Journal of the American Society of Nuclear Cardiology
# RESULTS : clinical images
Clinical images without DDMC (top row) and with DDMC (bottom row)
The motion trace for this case can be seen below this
Copyright American Society of Nuclear Cardiology

## Slide 6
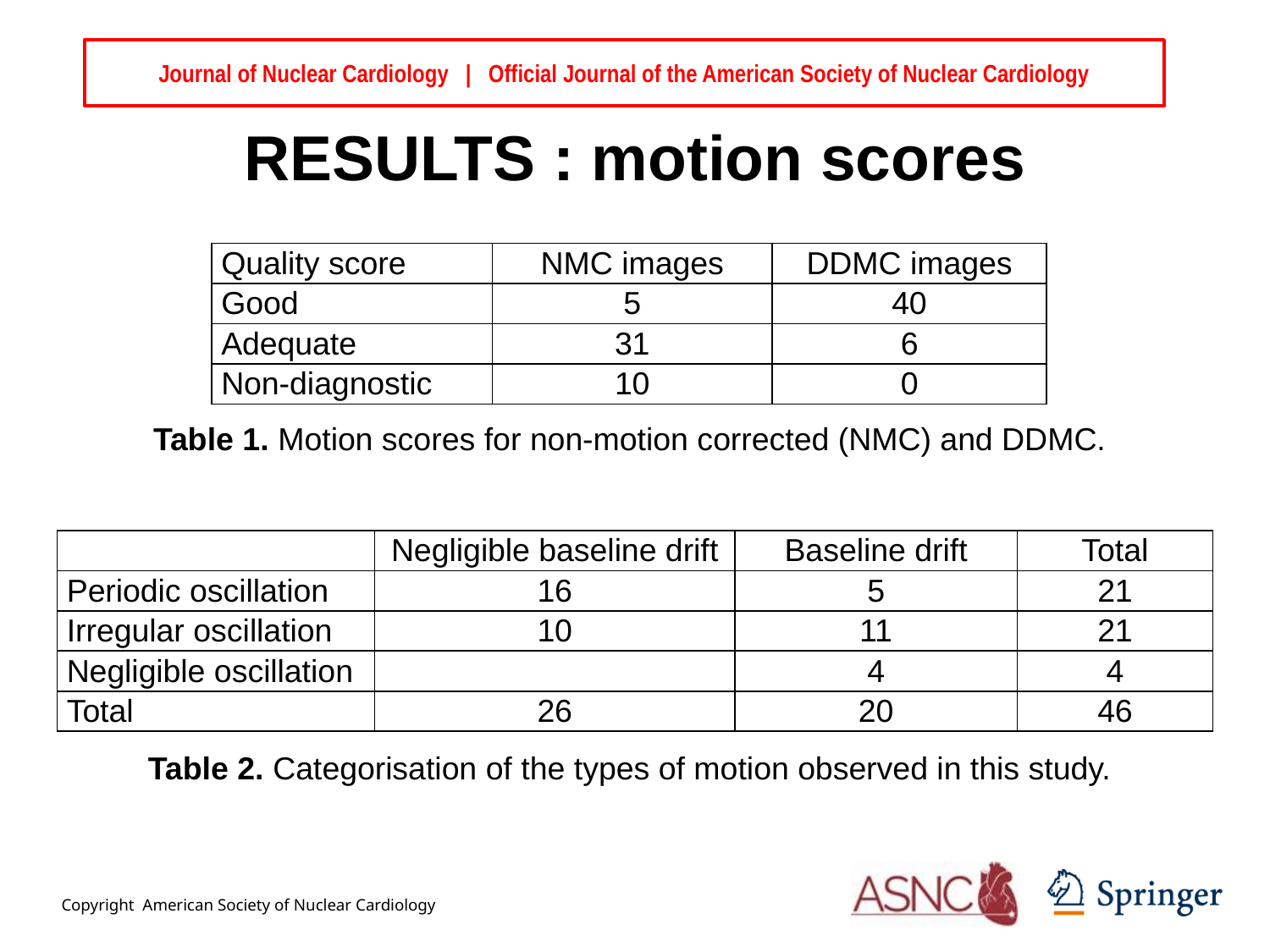

Journal of Nuclear Cardiology | Official Journal of the American Society of Nuclear Cardiology
# RESULTS : motion scores
| Quality score | NMC images | DDMC images |
| --- | --- | --- |
| Good | 5 | 40 |
| Adequate | 31 | 6 |
| Non-diagnostic | 10 | 0 |
Table 1. Motion scores for non-motion corrected (NMC) and DDMC.
| | Negligible baseline drift | Baseline drift | Total |
| --- | --- | --- | --- |
| Periodic oscillation | 16 | 5 | 21 |
| Irregular oscillation | 10 | 11 | 21 |
| Negligible oscillation | | 4 | 4 |
| Total | 26 | 20 | 46 |
Table 2. Categorisation of the types of motion observed in this study.
Copyright American Society of Nuclear Cardiology

## Slide 7
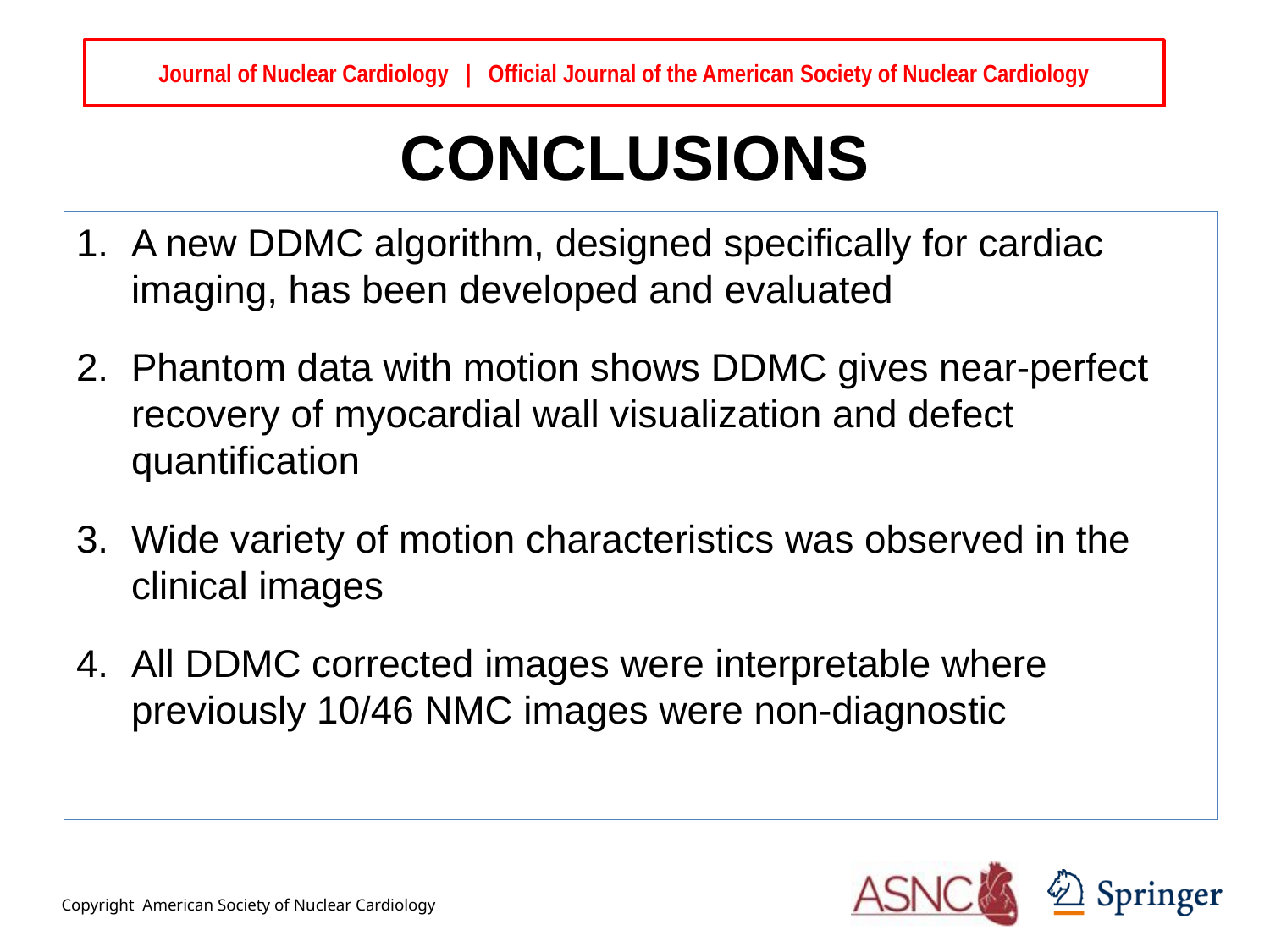

Journal of Nuclear Cardiology | Official Journal of the American Society of Nuclear Cardiology
# CONCLUSIONS
A new DDMC algorithm, designed specifically for cardiac imaging, has been developed and evaluated
Phantom data with motion shows DDMC gives near-perfect recovery of myocardial wall visualization and defect quantification
Wide variety of motion characteristics was observed in the clinical images
All DDMC corrected images were interpretable where previously 10/46 NMC images were non-diagnostic
Copyright American Society of Nuclear Cardiology
